# Guest editorial: From research design to the waiting room

**DOI:** 10.1016/j.qrmh.2025.100019

**Published:** 2025-07-30

**Authors:** Paula Hopeck

**Affiliations:** Associate Editor of QRMH, Associate Professor of Communication Studies, Commonwealth University of Pennsylvania at Bloomsburg, Bloomsburg, PA, USA

Earlier this year, at a doctor’s appointment, I heard another patient complain about the hospital-produced program playing in the waiting room: “I hate this [healthcare system] TV shit. It’s just patting themselves on the back.” Fast forward to last week, when I returned to the same healthcare facility. I passed a sign outside of the endoscopy unit that said, “Please do not eat food out of respect to the patients who are fasting.” The waiting room had the hospital-produced program on, including a segment featuring step-by-step instructions on how to prepare a delicious quinoa meal.

I imagine there are people like me who are grateful for the work that our healthcare teams do when we put our health in their hands. However, I imagine that there are also people like me who dread dealing with the inefficiencies of hospital systems. Particularly in the U.S., we are frustrated by a system that cannot help us immediately, makes us wait months for an appointment, rushes us through the appointment, and then subjects us to clips as my fellow patient said, of them patting themselves on the back. Sometimes, messages meant to celebrate and educate can come across as tone-deaf or irrelevant.

I acknowledge that a promotional video is probably from a marketing executive, not a direct healthcare provider. But it still highlights the problem that sometimes in the grand scheme of things, perhaps we lose touch with those that we are intending to serve.

As a health communication scholar, I understand the importance of educating and motivating patients, and a hospital waiting room is, in theory, a great place to do that. No distractions, patients must stay in the room, and patients can be reminded or encouraged to talk to their doctor about certain issues—seems like the perfect plan. As a patient, I am much more cynical. *Is it possible that I have this type of cancer? Could I be at risk for this illness? What if my insurance doesn’t cover this miracle treatment? What if my illness has progressed too much? Will I be able to take time off from work to get this extensive treatment? Why, after a day of fasting, do I have to watch this person talk about how delicious quinoa is?*

This issue’s three articles remind us of the importance of patients in healthcare research, from commentary on including patients in our own research projects and design, understanding the perspective of those living life after a stroke, and patient perceptions of physician assistants.

Elna Leth Pedersen, Hanne Agerskov, Torkell Ellingsen, and Connie Timmerman’s commentary, “How to Become Partners: Ways to Enhance the Quality of Patient and Public Involvement in Healthcare Research” navigates the challenges of involving “patient partners” in Patient and Public Involvement (PPI) research. Through their experiences of working with seven participants (or “co-thinkers”), the authors help researchers stay relevant for the audiences that we—hopefully—intend to help. This also means making research accessible using appropriate terminology in recruiting participants, effective motivation for becoming involved in research, which will inform how to craft an objective for the study.

Staci Defibaugh and Leah Onosato’s findings in “’They Just Don’t Have the “Doctor” in Front of Their Name:” Dimensions of Trust of Physician Assistants,” show the importance of the model of care that physician assistants (PAs) provide. Through 30 in-depth interviews with patients of PAs, the authors inform readers that PAs are knowledgeable and compassionate, and patients appreciate how PAs dedicate the time to listen to them. Patients feel heard by their PAs, believe in their abilities to provide the best quality care, and appreciate the space that PAs make for them. Defibaugh and Onosato acknowledge that this model can inform future directions in patient-centered care.

“Navigating the Life Stage after Stroke: From Life 2.0 to Stroke Prevention Models of Care—A Qualitative Exploration of Younger and Middle-Aged Adult Stroke Patients’ Experiences and Recommendations” by Sarah Ibrahim et al. utilizes Goffman’s dramaturgical theory to highlight the struggles and experiences of 12 individuals, 65 years of age and younger who have had a stroke or were at a higher risk of stroke. Using interviews as their data set, the authors craft a model that demonstrates the front stage, setting, backstage, and audience. Following a stroke, individuals face the challenge of changing their health habits, while also incorporating those changes into their professional and social lives. Among the several identified implications are incorporating peer-led interventions and exploring what is important to patients of this age group.

I hope that readers will be encouraged by these articles of the great research that adds to the ever-changing body of literature on advancing patient-and family-centered care. We are also reminded that patient experiences encompass so many aspects of our health research, from research design, to the bedside manner, to navigating life after a major health event, and back to the waiting room.

